# Heat stress compromises nutritional quality and flavor of bovine raw milk: Evidence from multi-omics analyses

**DOI:** 10.1016/j.fochx.2025.103361

**Published:** 2025-12-03

**Authors:** Yuchao Zhao, Fenghong Wang, Ying Wang, Jian Tan, Haoyu Niu, Gang Guo, Luoyun Fang, Linshu Jiang

**Affiliations:** a Beijing Key Laboratory of Dairy Cow Nutrition, College of Animal Science and Technology, Beijing University of Agriculture, Beijing 102206, China; b Sano (Yangling) Modern Animal Nutrition Co., LTD, Yangling 712100, China; c Beijing Sunlon Livestock Development Co., Ltd., Beijing 100076, China

**Keywords:** Antioxidant capacity, Dairy cows, Heat stress, Milk quality, Metabolomics, Flavor compounds

## Abstract

Heat stress is a growing concern for dairy production under global climate change. This study employed integrated multi-omics approaches to investigate how heat stress affects the antioxidant capacity, microbiota, metabolite profiles, lipid composition, and flavor compounds in raw milk. Results revealed that heat stress reduced antioxidant levels and altered the milk microbiome, favoring potentially spoilage-associated bacteria. Metabolomic analysis indicated disrupted energy, amino acid, and lipid metabolism, with reductions in beneficial unsaturated fatty acids, conjugated linoleic acid, and polar lipids such as phosphatidylcholine and sphingomyelin. Notably, several off-flavor volatiles, including hexanal, ketones, and sulfur compounds, increased in heat-stressed milk, while sweet esters declined. These compositional and sensory changes may compromise milk quality, nutritional value, and consumer acceptability. This study provides a comprehensive biochemical basis for understanding how heat stress affects milk quality, offering important references for quality assessment and risk monitoring in dairy production under warming climates.

## Highlights

• Heat stress significantly alters milk's chemical and microbial composition.
• Psychrotrophic bacterial abundance increases in heat-stressed milk.
• Heat-stressed milk shows reduced antioxidant capacity and amino acids.
• Off-flavor compounds increase in milk under heat stress.
• Unsaturated fatty acids and polar lipids decrease under heat stress.

## 1 Introduction

With the intensification of global climate change, episodes of heat stress are becoming more frequent and severe, increasingly jeopardizing dairy productivity and milk safety (Nardone et al., 2010). Dairy cattle are particularly vulnerable to thermal stress, and prolonged exposure to elevated temperatures during summer can significantly reduce milk production and alter its composition (McWhorter et al., 2023). For example, heat stress often leads to declines in milk protein and fat percentages, which highlights the more pronounced impact of heat stress on milk quality than on milk volume (Chen et al., 2024; Moore et al., 2023). These heat-induced losses not only cause economic damage but also raise concerns about sustaining a safe and nutritious milk supply in a warming climate. Indeed, rising temperatures have been linked to increased microbial contamination risks in raw milk and higher incidence of mastitis (Gayathri et al., 2025), underscoring the food safety challenges associated with heat stress in dairy herds.

Maintaining milk quality is critical because milk is a cornerstone of human nutrition worldwide. As a nutrient-dense food, milk delivers essential amino acids, bioavailable proteins, and critical vitamins and minerals—particularly calcium—contributing significantly to human nutrition (Yang, Meng, et al., 2025). It is recognized as a nutritionally dense food that supports bone health and overall dietary quality. The dairy sector's contributions are significant – for instance, milk is the leading global source of calcium and a major provider of B vitamins and high-quality proteins (Aslanhan et al., 2024). Any decline in milk quality (such as reduced protein content or altered fatty acid profile) can diminish its nutritional value for consumers. Moreover, heat-stressed cows may produce milk with higher somatic cell counts or microbial loads, potentially affecting shelf life and safety (Fan et al., 2019).

Furthermore, heat-stressed cows experience oxidative stress due to increased production of reactive oxygen species coupled with depleted antioxidant defenses (Akköse & Vural, 2025; Li et al., 2021). This oxidative imbalance can affect cellular health and might influence the milk's content of oxidative markers or vitamins. A recent study noted that heat stress significantly modulated milk fat content and altered plasma metabolite profiles in cows (Alberghina et al., 2024), suggesting that the chemical constituents of milk (such as fatty acids and metabolites) are shifted under thermal stress. Changes in cow behavior and rumen function during heat stress (e.g., reduced feed intake and altered ruminal fermentation) can also lead to differences in milk's flavor and aroma compounds (Yang, Wu, et al., 2025). For instance, metabolic byproducts like ketones or free fatty acids may accumulate and impart off-flavors to the milk when cows are in negative energy balance or oxidative stress (Chin et al., 2024; Moore et al., 2024; Yan et al., 2025). Although subtle, such sensory changes could impact consumer acceptance of dairy products. Overall, these findings highlight that milk quality is a multifaceted trait– encompassing nutritional, microbiological, and sensory aspects – all of which may be influenced by environmental heat (Bilal et al., 2024; Steinshamn et al., 2014; Tadesse et al., 2024). Ensuring the integrity of milk's nutritional and safety attributes under climate stress is therefore paramount for public health and food security.

To comprehensively understand how summer heat stress alters milk composition, multi-omics approaches offer a powerful solution. By integrating data across genomic, transcriptomic, proteomic, and metabolomic levels, multi-omics enables a systems-level understanding of biological responses. By simultaneously quantifying hundreds of metabolites, lipids, and other molecules, researchers can gain valuable insights into complex physiological responses that would be missed by single-measure analyses. The present study employs an integrated multi-omics approach to investigate the impact of summer heat stress on milk quality in Holstein dairy cows. We combine metabolomics, lipidomics, 16S rRNA gene sequencing, and flavor compound analysis to holistically characterize changes in milk composition under heat stress. Our findings will shed light on the resilience of milk composition to environmental stress and identify potential biomarkers of heat stress in dairy cows, ultimately contributing to sustainable dairy production and food quality assurance under warming climatic conditions.

## 2 Materials and methods

### 2.1 Use of animals

All experimental procedures involving animals complied with the Regulations for the Administration of Affairs Concerning Experimental Animals of the People's Republic of China were approved by the Institutional Animal Care and Use Committee at Beijing University of Agriculture (Approval No. BUA2023282, date: Jan 4th, 2023). The study adhered to the animal research guidelines by Beijing University of Agriculture. Dairy cows used in this study were sourced from a commercial dairy farm in Beijing, China. Twenty-four cows were all clinically healthy, second-parity Holstein dairy cows, and aged between 2 and 4 years. The cows were not subjected to any procedures that involved euthanasia or slaughter, and all efforts were made to minimize their discomfort during the study.

### 2.2 Experimental design and sampling

This study was carried out on a commercial dairy farm in Beijing, China, with the objective of assessing how summer heat stress influences raw milk quality in lactating cows through a multi-omics analysis. A total of 24 clinically healthy Chinese Holstein cows in mid-lactation were enrolled in the study. All animals were second-parity, and their days in milk (DIM) ranged between 130 and 150 days to ensure physiological consistency across groups. The cows were divided into two groups based on seasonal temperature conditions. The heat stress-free (HS-free) group, consisting of 12 cows, was sampled in mid-April when the average ambient temperature stayed within the thermoneutral zone (temperature-humidity index [THI] < 68). The heat stress (HS) group, also with 12 cows, was sampled in mid-July after a period of sustained high temperatures and humidity, with the THI exceeding 78 for at least seven consecutive days, indicating mild to moderate heat stress. A summary of cow characteristics is provided in Supplemental Table S1.

All animals were housed in a closed-type free-stall barn equipped with natural ventilation. This controlled housing environment was selected to minimize the influence of photoperiod differences between seasons on the cows' metabolic status. The cows were managed under the same routine throughout the study. Cows were provided a total mixed ration formulated to meet or slightly exceed their nutritional needs as outlined by NASEM (2021), and were fed twice daily at 0800 and 1730 h with ad libitum access. Water was available ad libitum, and milking was performed three times daily at 0600, 1400, and 2100 h. No feed changes, management modifications, or veterinary interventions occurred during the experimental period. The composition and nutritional content of the diets are detailed in Supplemental Table S2.

To minimize circadian and postprandial effects, milk samples were collected from all cows between 0600 and 0800 h during the morning milking session, after thorough udder cleaning and prior to feeding. Approximately 200 mL of milk was collected per cow from all functional quarters, pooled, and immediately divided into aliquots for downstream analyses (Fan et al., 2019).

### 2.3 Milk antioxidant activity analysis

Milk antioxidant capacity was assessed by quantifying total antioxidant capacity (T-AOC), and the enzymatic activities of superoxide dismutase (SOD), glutathione peroxidase (GSH-Px), and catalase (CAT), along with malondialdehyde (MDA) concentration. Milk samples were thawed at room temperature and processed following the protocol of Zhao et al. (2013) with slight adjustments. Samples were initially centrifuged at 14,000 ×*g* for 30 min at 4 °C to remove fat. The supernatant was then treated with 4 % acetic acid to precipitate casein and centrifuged again under the same conditions. The resulting clarified solution was used for antioxidant assays.

Commercial kits from Beijing Solarbio Science & Technology Co., Ltd. (Beijing, China) were utilized to measure each parameter: T-AOC (#BC1315), SOD (#BC5165), GSH-Px (#BC1195), and CAT (#BC0205)), following the supplier's instructions. Milk MDA was analyzed using the commercial kit (#AKFA013M, Beijing Boxbio Science ＆ Technology Co.,Ltd., Beijing, China). T-AOC was evaluated using the ferric-reducing antioxidant power method. SOD activity was determined via the xanthine oxidase approach, while GSH-Px was assessed based on the rate of glutathione oxidation. CAT activity was measured by monitoring the decomposition of H₂O₂, and MDA content was determined using the TBARS (thiobarbituric acid reactive substances) assay. All measurements were conducted in triplicate using a microplate reader (SpectraMax iD3, Molecular Devices, USA). Enzyme activities were reported as U/mL and MDA as μmol/L.

### 2.4 Milk microbiota profiling via 16S rRNA gene sequencing

Milk microbial communities were characterized via 16S rRNA gene amplicon sequencing. To extract bacterial DNA, 2 mL of thawed milk was centrifuged at 14,000 ×*g* for 20 min at 4 °C to collect microbial pellets. These pellets were rinsed twice with sterile phosphate-buffered saline (PBS, pH 7.4), and DNA was subsequently extracted using the E.Z.N.A.® Soil DNA Kit (Omega Bio-Tek, Norcross, GA, USA), with slight modifications to improve cell disruption. The quantity and purity of extracted DNA were evaluated using a NanoDrop 2000 spectrophotometer (Thermo Fisher Scientific, USA).

The V3–V4 regions of the bacterial 16S rRNA gene were amplified using the universal primers 341F (5′-CCTACGGGNGGCWGCAG-3′) and 806R (5′-GGACTACHVGGGTATCTAAT-3′). Amplified products were cleaned with the Agencourt AMPure XP beads (Beckman Coulter, USA), quantified via a Qubit fluorometer (Thermo Fisher Scientific), and then pooled in equimolar concentrations. Sequencing was conducted on an Illumina MiSeq platform (Illumina, San Diego, CA, USA) with paired-end 2 × 300 bp reads, following standard operating procedures.

Raw sequencing data were processed using the QIIME2 software package (version 2021.2). In summary, demultiplexing of paired-end reads was followed by quality filtering, denoising, and amplicon sequence variant (ASV) identification via the DADA2 algorithm. Potential chimeric reads were detected and excluded. Taxonomic classification was carried out using a naïve Bayes classifier trained on the SILVA 138 reference database. Diversity metrics, including alpha and beta diversity, along with taxonomic distributions at various levels, were computed within the QIIME2 framework. All raw sequencing data generated in this study have been submitted to the Genome Sequence Archive (GSA: CRA027878) at the National Genomics Data Center, part of the Chinese Academy of Sciences.

### 2.5 Milk fatty acid composition analysis by GC-FID

Milk fatty acids were analyzed as fatty acid methyl esters (FAMEs) using gas chromatography with a flame ionization detector (FID), based on the modified procedure of Wang, Chen, et al. (2022). In brief, 2 mL of thawed milk was treated with 2 mL ammonium hydroxide (≥25 %), 1 mL ethanol (≥95 %), and 50 mg pyrogallic acid, followed by incubation in a 70 °C water bath for 20 min to hydrolyze milk lipids. After cooling, 4 mL of a n-hexane/isopropanol mixture (3:2, *v*/v) was added, vortexed for 30 s, and centrifuged at 10,000 ×*g* for 5 min at 4 °C. The upper organic layer, containing extracted milk fat, was separated for methylation. To prepare FAMEs, the fat extract was dissolved in 2 mL n-hexane, then reacted with 2 mL of 2 % methanolic sodium hydroxide at 50 °C for 20 min. Next, 2 mL of 10 % acetyl chloride in methanol was added, and the reaction proceeded at 80 °C for 2.5 h. Upon cooling, 3 mL n-hexane and 3 mL ultrapure water were added, and the upper hexane layer was recovered. Anhydrous sodium sulfate (0.5 g) was used to remove any remaining moisture before transferring the final solution to GC vials for analysis.

FAME profiles were analyzed using a 7820 A gas chromatograph (Agilent Technologies, USA) equipped with a flame ionization detector (FID), a temperature-programmed injector, and a CP-Sil 88 capillary column (100 m × 0.25 mm × 0.20 μm; Agilent Technologies). Hydrogen served as the carrier gas at a constant flow rate of 1 mL/min. The injector and detector temperatures were maintained at 270 °C and 300 °C, respectively. The oven temperature program was as follows: initial hold at 60 °C for 4 min, then ramped at 2 °C/min to 200 °C with a 30-min hold, followed by a second ramp at 2 °C/min to 240 °C, and held for a final 19 min. FAMEs were identified by comparing their retention times with those of reference standards, including a 37-component FAME mix (Solarbio, Beijing, China) and specific isomers of octadecadienoic acid methyl esters (*cis*-9, *trans*-11 and *trans*-10, *cis-*12; Sigma-Aldrich, USA). Quantification was based on external standard calibration, and results were reported as grams per 100 g of total fatty acids. Each sample was analyzed in triplicate to ensure accuracy. A chromatographic profile of milk fatty acids included in the method is presented in Supplementary Materials Fig. S1.

### 2.6 LC–MS-based lipidomics analysis

Lipidomic analysis of milk samples was performed using liquid chromatography–mass spectrometry (LC–MS). In brief, 100 μL of thawed milk was combined with 400 μL chilled methanol and 800 μL methyl tert-butyl ether (MTBE) in a 2 mL microcentrifuge tube. The mixture was vortexed for 30 s and left at room temperature for 10 min to promote lipid extraction and phase separation. Next, 200 μL of ultrapure water was added, followed by a second vortex and centrifugation at 14,000 ×*g* for 10 min at 4 °C. The upper, lipid-rich organic layer was carefully collected and evaporated to dryness under a stream of nitrogen. Finally, the dried residue was dissolved in 100 μL of isopropanol/methanol (1:1, *v*/v) for subsequent LC–MS detection.

Lipidomic profiling was carried out using an ultra-high-performance liquid chromatography (UHPLC) system (Vanquish, Thermo Fisher Scientific, USA) coupled with a Q Exactive Plus Orbitrap mass spectrometer (Thermo Fisher Scientific, USA). Separation was performed on a Hypersil GOLD C18 column (100 × 2.1 mm, 1.9 μm particle size). The mobile phase consisted of solvent A (acetonitrile:water, 60:40 v/v) and solvent B (isopropanol:acetonitrile, 90:10 v/v), both containing 10 mM ammonium formate and 0.1 % formic acid. The flow rate was maintained at 0.3 mL/min. The elution gradient started with 30 % B (0–2 min), ramped from 30 % to 100 % B over 2–25 min, held at 100 % B from 25 to 30 min, dropped back to 30 % B at 30.1 min, and was re-equilibrated at 30 % B from 30.1 to 35 min. To ensure data quality and instrument stability, pooled quality control samples composed of aliquots from all test samples were injected after every eight sample runs.

Mass spectrometric detection was carried out in both positive and negative electrospray ionization (ESI) modes. Instrument parameters were set with a capillary temperature of 320 °C, spray voltages of 3.5 kV for positive mode and − 2.8 kV for negative mode. The full scan range was set from *m*/*z* 200 to 2000, using a resolution of 70,000 (FWHM). Data-dependent MS/MS acquisition was applied to enable structural identification of lipid molecules. Lipid species were identified and quantified using LipidSearch version 4.2 (Thermo Fisher Scientific, USA), integrating exact mass, chromatographic retention behavior, and tandem MS spectral features. Representative chromatograms shown in supplementary material Fig. S2.

### 2.7 LC–MS-based metabolomics analysis

Untargeted metabolomics of milk samples was conducted via LC–MS. To extract metabolites, 100 μL of thawed milk was mixed with 400 μL of chilled methanol and vortexed for 30 s. The mixture was then incubated at −20 °C for 30 min to facilitate protein precipitation. After incubation, samples were centrifuged at 14,000*g* for 15 min at 4 °C, and the resulting supernatant was transferred to a new vial. The collected supernatants were evaporated to dryness using a vacuum concentrator. Finally, dried residues were redissolved in 100 μL of a 50:50 methanol–water solution and subjected to LC–MS analysis.

LC–MS analysis was carried out using a Vanquish UHPLC system coupled with a Q Exactive Plus Orbitrap mass spectrometer (Thermo Fisher Scientific, USA) featuring an ESI interface. Separation of metabolites was performed on a Hypersil GOLD C18 column (100 mm × 2.1 mm, 1.9 μm). The mobile phase consisted of solvent A (water with 0.1 % formic acid) and solvent B (acetonitrile with 0.1 % formic acid), delivered at a flow rate of 0.3 mL/min. A 5 μL sample volume was injected. The gradient elution program was as follows: 0–2 min, 5 % B; 2–15 min, ramping to 95 % B; 15–17 min, 95 % B; 17–17.1 min, return to 5 % B; 17.1–20 min, equilibration at 5 % B.

Mass spectrometric detection was performed in both positive and negative ESI modes. Data were acquired over an *m*/*z* range of 100–1500. The resolution was set to 70,000 for full MS scans and 17,500 for MS/MS scans. Source parameters included a capillary temperature of 320 °C, with spray voltages of 3.5 kV (positive) and − 2.8 kV (negative). Examples of chromatograms are reported in Fig. S3 in Supplementary materials.

The raw LC–MS datasets were analyzed using Progenesis QI software (Waters, UK) for data preprocessing steps, including peak detection, alignment, noise reduction, and signal normalization. Metabolite identification was carried out by matching accurate mass, retention time, and MS/MS fragmentation profiles with entries in public metabolite databases such as HMDB, METLIN, and KEGG. Tentative metabolite assignments were made by evaluating spectral similarity and consistency in chromatographic behavior. Prior to downstream statistical analysis, data were normalized to total ion current (TIC) and subjected to quality control filters to ensure reliability and reproducibility.

### 2.8 Determination of amino acids in milk

Approximately 100 mg of freeze-dried milk powder was carefully placed into a hydrolysis tube, ensuring minimal contact with the inner wall. Subsequently, 4 mL of 6 mol/L HCl was added, and nitrogen gas was introduced for 15 min to displace oxygen and prevent oxidative degradation. The sealed tube was then subjected to hydrolysis at 110 °C for 22–24 h in a thermostatic oven. After cooling to room temperature, the contents were transferred into a 100 mL volumetric flask and diluted with ultrapure water.

A 2 mL portion of the hydrolysate was evaporated to dryness under nitrogen at 60 °C and reconstituted in 2 mL of 0.02 mol/L HCl. The solution was filtered through a 0.22 μm membrane filter prior to analysis. Amino acid profiling was performed using a Hitachi L-8900 automatic amino acid analyzer equipped with a sodium cation exchange column (4.6 mm × 60 mm, 3 μm, packed with 2622 resin). Separation was achieved using a gradient of sodium citrate buffers (pH 3.0–4.9) at a flow rate of 0.4 mL/min. Column and post-column reaction temperatures were maintained at 55 °C and 135 °C, respectively. Detection was based on post-column derivatization with ninhydrin, with absorbance measured at 570 nm for most amino acids and 440 nm for proline. Peak identification and quantification were carried out by comparing retention times and peak areas to known standards.

### 2.9 Milk volatile metabolomics analysis using HS-SPME/GC–MS

Volatile compounds in milk were analyzed using headspace solid-phase microextraction combined with gas chromatography–mass spectrometry (HS-SPME/GC–MS), following a modified procedure based on Zhao et al. (2023a). For analysis, 10 mL of raw milk was placed into a 20 mL headspace vial and spiked with 20 μL of an internal standard solution containing n-octadecane (3.6 mg/mL). The vial was sealed using a PTFE-lined cap and incubated in a water bath at 40 °C with constant agitation for 30 min to facilitate headspace equilibrium of volatiles.

A 50/30 μm DVB/CAR/PDMS fiber (Supelco, Bellefonte, PA, USA) was used to extract volatile organic compounds from the headspace. The SPME fiber was exposed to the headspace for 30 min to adsorb the volatiles. Following extraction, the fiber was inserted into the GC injector and desorbed at 250 °C for 5 min under splitless conditions to release the absorbed analytes for chromatographic separation and identification.

Volatile compound analysis was performed using a GC–MS system (7890 A–5975C; Agilent Technologies, USA) equipped with a DB-WAXETR capillary column (30 m × 0.32 mm ID, 0.25 μm film thickness; Agilent). The injection port was maintained at 250 °C and operated in splitless mode to ensure complete sample transfer into the column. Chromatographic separation followed this oven program: initial hold at 40 °C for 5 min, then ramping at 5 °C/min to 250 °C, with a final hold of 10 min. Helium served as the carrier gas at a constant flow of 1.7 mL/min. The electron ionization (EI) source was set at 230 °C with 70 eV ionization energy. The interface and quadrupole temperatures were maintained at 280 °C and 150 °C, respectively. Data acquisition was carried out in full scan mode across a mass range of 25–450 amu. A chromatographic profile of milk volatile compounds included in the method is presented in Supplemental Fig. S4.

Raw spectral data were analyzed using MassHunter Workstation Quantitative Analysis software (version 10.0.707.0, Agilent Technologies). The software automatically extracted retention times, peak areas, and *m*/*z* values through its integrated deconvolution and quantification functions. Compound identification was achieved by matching mass spectra and calculated retention indices against reference databases, including the NIST Mass Spectral Library and MS-DIAL. Only compounds with spectral match scores exceeding 80 % were considered reliable. Furthermore, only metabolites consistently detected in at least 80 % of the samples within each treatment group were retained for statistical analysis.

### 2.10 Statistical analysis

Comparisons of dry matter intake, milk yield and components, antioxidant indicators, amino acid, and fatty acid profiles were conducted using one-way ANOVA in SAS software (version 9.4; SAS Institute Inc., Cary, NC, USA), with heat stress condition (HS vs. HS-free) defined as the fixed factor. Prior to analysis, data normality and homoscedasticity were assessed via the Shapiro–Wilk and Levene's tests, respectively. When significant effects were detected (*P* < 0.05), Tukey's multiple comparisons test was employed.

For lipidomic, metabolomic, and volatile compound datasets, multivariate statistical approaches including principal component analysis (PCA) and orthogonal partial least squares discriminant analysis (OPLS-DA) were performed using the ropls package in R and SIMCA software (v14.1). Discriminative metabolites or lipids were screened based on variable importance in projection (VIP > 1.0) and false discovery rate (FDR)-adjusted *P* < 0.05.

Microbial diversity metrics were evaluated within the QIIME2 platform. Differences in alpha diversity and relative taxonomic abundance were tested using Wilcoxon rank-sum tests, while beta diversity was compared by permutational multivariate analysis of variance (PERMANOVA) based on Bray–Curtis dissimilarities with 999 permutations. Taxa with FDR-adjusted *P*-values <0.05 were considered significantly different between groups.

## 3 Results

### 3.1 Environmental conditions, cow physiological and production data

Supplemental Tables S3–S5 offer additional insights into environmental conditions (temperature and relative humidity), physiological parameters (rectal temperature and respiration rate), and milk production data. The THI varied between 70.2 and 87.90 in the HS group, indicating that the cows experienced mild to moderate heat stress. Notably, the HS group showed significantly higher rectal temperature and respiratory rate, along with reduced dry matter intake, milk yield, protein content, and altered levels of milk urea nitrogen and somatic cell count compared to the HS-free group (*P* < 0.05).

### 3.2 Milk antioxidant capacity

The antioxidant capacity parameters in milk differed significantly between the HS-free and HS cows (Table 1). Cows exposed to heat stress exhibited substantial reductions in total antioxidant capacity (T-AOC, *P* = 0.003), superoxide dismutase (SOD, *P* = 0.005), glutathione peroxidase (GSH-Px, *P* = 0.012), and catalase (CAT, *P* = 0.021) compared to cows not under heat stress. Conversely, malondialdehyde (MDA) levels, a marker of lipid peroxidation, were significantly elevated in the HS group (*P* = 0.036), reflecting increased oxidative damage induced by thermal stress.

**Table 1:** Comparison of milk antioxidant capacity parameters between HS-free and HS groups.

Item^1^	Group^2^		SEM	*P*-value
	HS-free	HS		
T-AOC, U/mL	8.28	6.13	0.409	0.003
SOD, U/mL	115.0	82.9	4.53	0.005
GSH-Px, U/mL	383	325	12.3	0.012
CAT, U/mL	13.91	9.90	1.114	0.021
MDA, ng/mL	2.04	3.83	0.480	0.036

1 T-AOC, total antioxidant capacity; SOD, superoxide dismutase; GSH-Px, glutathione peroxidase; CAT, catalase; MDA, malondialdehyde.

2 HS-free, heat stress-free; HS, heat stress.

### 3.3 Milk microbiota composition

A total of 1,141,321 raw sequences were generated from the milk microbiota analysis, with an average read length of 425 bp. Following quality filtering procedures—including trimming, denoising, and removal of chimeric reads—1,018,798 high-quality sequences remained, averaging 42,450 reads per milk sample. These sequences were grouped into 682 distinct amplicon sequence variants (ASVs).

**Fig. 1:**
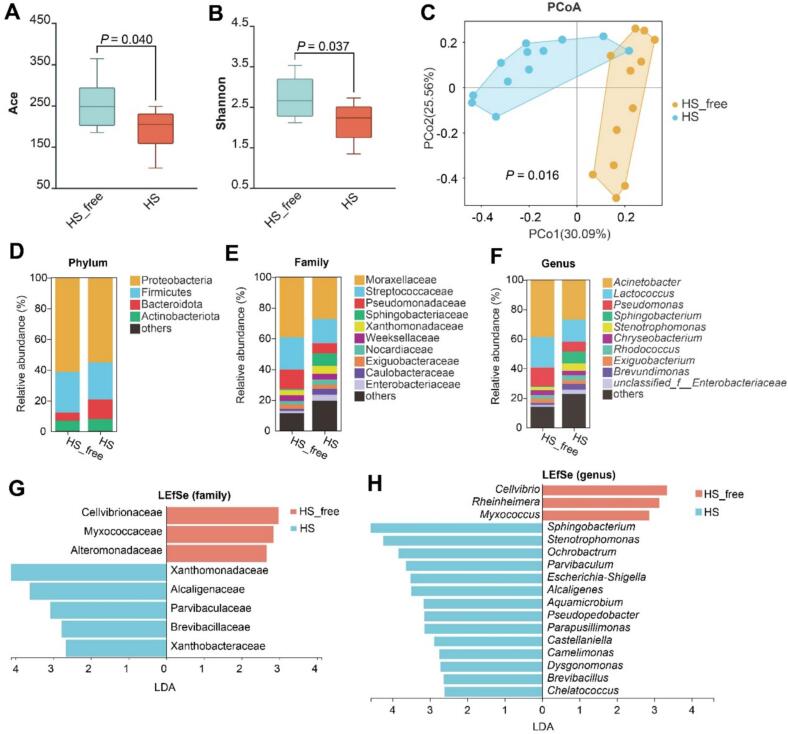
Effects of heat stress on milk microbiota diversity and composition. (A, B) Alpha diversity indices (Ace and Shannon) of milk microbiota were significantly reduced in the HS group compared to the HS-free group (*P* < 0.05). (C) Principal coordinate analysis (PCoA) based on Bray–Curtis distances showed distinct clustering between the two groups (*P* = 0.016). (D–F) Relative abundance of microbial taxa at the phylum (D), family (E), and genus (F) levels between HS-free and HS groups. (G, H) LEfSe analysis identified differentially enriched bacterial taxa at the family (G) and genus (H) levels, with LDA score > 2.0. HS, heat stress; HS-free, heat stress-free.

Analysis of alpha diversity showed that microbial richness and diversity were significantly diminished in the HS group. Specifically, both the Ace and Shannon indices were lower in the HS group compared to the HS-free group (*P* = 0.040 and *P* = 0.037, respectively; Fig. 1A, B). Moreover, PCoA using Bray–Curtis metrics demonstrated clear group separation (ANOSIM, *P* = 0.016; Fig. 1C), suggesting that heat stress substantially reshaped the milk microbial community composition. Fig. 1D–F illustrates the overall microbial composition at the phylum, family, and genus levels. Proteobacteria was the predominant phylum in both groups, followed by Firmicutes, Bacteroidota, and Actinobacteriota. At the family level, Moraxellaceae, Streptococcaceae, and Pseudomonadaceae were relatively abundant. The dominant genera included *Acinetobacter*, *Lactococcus*, and *Pseudomonas*.

Differential abundance analysis using LEfSe (Fig. 1G,H) revealed that members of the families Cellvibrionaceae, Myxococcaceae, and Alteromonadaceae, as well as the genera *Cellvibrio*, *Rheinheimera*, and *Myxococcus*, were significantly enriched in the HS-free group. In contrast, *Sphingobacterium*, *Stenotrophomonas*, *Ochrobactrum*, *Parvibaculum*, and *Escherichia-Shigella* were more abundant in the HS group.

### 3.4 Milk untargeted metabolome profiles and targeted amino acid profiles

**Fig. 2:**
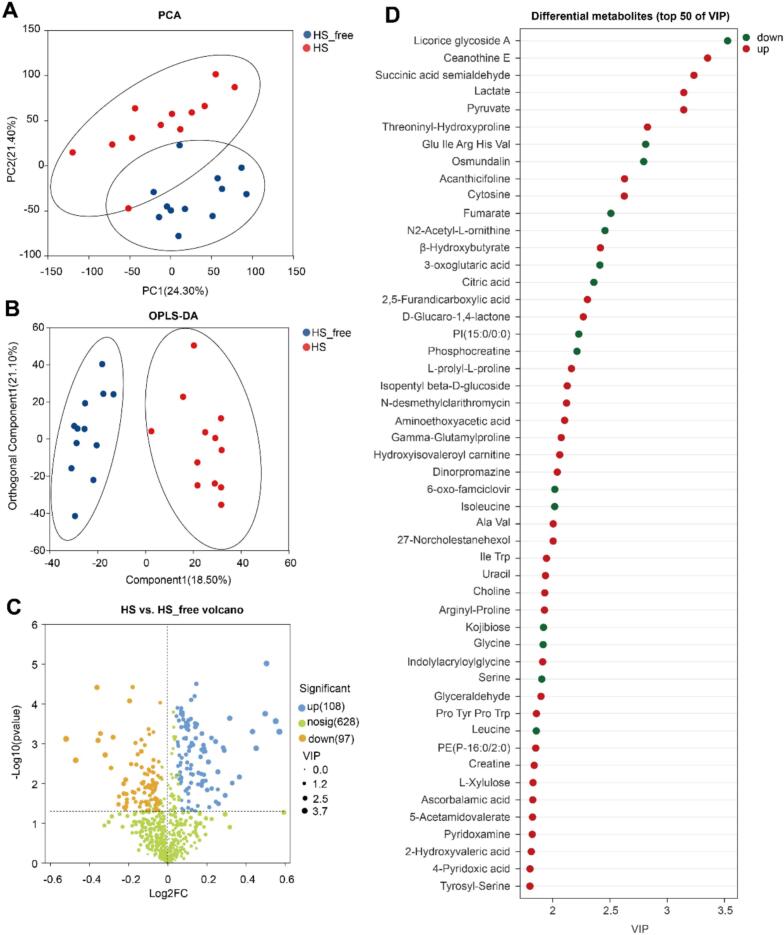
Non-targeted metabolomics analysis of milk samples from HS and HS-free cows. (A) Score plots for a principal component analysis (PCA) model. (B) Score plots of orthogonal partial least squares-discriminant analysis (OPLS-DA) for the milk metabolite profiles. (C) Volcano plots of metabolites in milk. (D) Top 50 discriminative metabolites ranked by variable importance in projection (VIP) scores from the OPLS-DA model. Red and green dots represent upregulated and downregulated metabolites in HS cows, respectively. PCA, principal component analysis; OPLS-DA, orthogonal partial least squares-discriminant analysis; VIP, variable importance in projection; HS, heat stress; HS-free, heat stress-free. (For interpretation of the references to color in this figure legend, the reader is referred to the web version of this article.)

Our untargeted metabolomics workflow quantified 1003 milk features, after normalization and removal of background and false positives. Out of these, 833 milk metabolites were annotated. To investigate the metabolic alterations in milk induced by heat stress, a non-targeted metabolomics analysis was conducted. The PCA score plot showed a clear separation between the HS and HS-free groups (Fig. 2A), indicating distinct global metabolic profiles. This separation was further confirmed by OPLS-DA, with an obvious discrimination between the two groups (Fig. 2B), suggesting that heat stress significantly altered the milk metabolome. A total of 205 metabolites exhibited significant differences between the two groups based on volcano plot analysis, with 108 showing increased levels and 97 showing decreased levels in the HS group (Fig. 2C).

Among the top 50 discriminative features (ranked by VIP scores), (Fig. 2D), several belonged to key metabolic categories, including energy metabolism (e.g., lactate, pyruvate, β-hydroxybutyrate, fumarate, citric acid, phosphocreatine), amino acid metabolism (e.g., isoleucine, glycine, serine, leucine), and nucleotide and cofactor metabolism (e.g., cytosine, uracil, pyridoxamine, creatine). Specifically, the HS group showed increased levels of lactate, pyruvate, β-hydroxybutyrate, cytosine, uracil, creatine, and pyridoxamine, while fumarate, citric acid, phosphocreatine, and several amino acids including isoleucine, glycine, serine, and leucine were significantly decreased.

Heat stress led to notable changes in the levels of multiple amino acids in milk (Table 2). Compared with the HS-free group, the HS group exhibited decreased levels of most essential amino acids (EAA), including Arg, Thr, Val, Met, Ile, Leu, Lys (all *P* < 0.05), resulting in a lower total EAA concentration (*P* = 0.044). Among non-essential amino acids (NEAA), Glu, Gly, Ala, and Ser were significantly reduced in HS cows (*P* < 0.05), with the lower total NEAA (*P* = 0.046). As a result, concentrations of both branched-chain amino acids (BCAA) and total amino acids (TAA) were significantly lower in the HS group (*P* = 0.043 and *P* = 0.042, respectively), reflecting a general reduction in amino acid availability in milk due to heat stress.

**Table 2:** Amino acid concentration in milk of HS-free and HS dairy cows.

Item^1^	Group^2^		SEM	*P*-value
	HS-free	HS		
*EAA (μmol/L)*				
Arg	1.81	1.55	0.132	0.045
Thr	3.40	2.91	0.187	0.033
Val	5.11	4.47	0.190	0.041
Met	1.63	1.27	0.106	0.044
Ile	3.60	3.00	0.135	0.048
Leu	7.11	6.08	0.228	0.046
Phe	2.60	2.25	0.154	0.072
Lys	5.30	4.60	0.135	0.047
His	1.65	1.43	0.108	0.075
Subtotal	32.21	27.56	1.890	0.044
*NEAA (μmol/L)*				
Cys	0.25	0.21	0.020	0.084
Glu	15.72	13.16	0.350	0.040
Tyr	2.46	2.09	0.135	0.088
Gly	2.59	2.10	0.124	0.033
Ala	3.58	3.05	0.090	0.042
Ser	5.17	4.40	0.171	0.042
Pro	5.86	4.52	0.310	0.083
Asp	5.60	4.90	0.325	0.054
Subtotal	41.23	34.43	2.236	0.046
BCAA	15.82	13.55	0.334	0.043
TAA	73.44	61.99	2.921	0.042

1 EAA, essential amino acid; NEAA, nonessential amino acids; BCAA, branched chain amino acids; TAA, total amino acids.

2 HS-free, heat stress-free; HS, heat stress.

### 3.5 Lipid profiles and fatty acids

Significant alterations in milk fatty acid composition were observed between cows in the HS-free and HS conditions (Table 3). The HS group exhibited higher proportions of saturated fatty acids, such as C14:0 (*P* = 0.012) and C16:0 (*P* = 0.024), while the proportions of C4:0 (*P* = 0.043) and C12:0 (*P* = 0.019) were also affected. For unsaturated fatty acids, the HS group showed reduced levels of C18:1 *cis*-9 (*P* = 0.015) and C18:2 *cis*-9, *trans*-11 (*P* < 0.001). Additionally, the proportion of C18:3n-3 was lower in the HS group compared with the HS-free group (*P* = 0.031). When fatty acids were classified by group, heat stress significantly increased total saturated fatty acids (*P* = 0.014) while decreasing total monounsaturated fatty acids (MUFA) (*P* = 0.011). No significant differences were observed in total polyunsaturated fatty acids (PUFA) (*P* = 0.169) or total conjugated linoleic acids (CLA) content (*P* = 0.135) between the two groups.

**Table 3:** Individual fatty acids (g/100 g total fatty acids) in milk of HS-free and HS dairy cows.

Item^1^	Group^2^		SEM	*P*-value
	HS-free	HS		
*FA proportions, % of total FA*				
C4:0	1.95	1.76	0.088	0.043
C6:0	1.03	0.92	0.091	0.519
C8:0	1.43	1.41	0.093	0.177
C10:0	3.39	3.15	0.359	0.625
C12:0	3.24	3.68	0.153	0.019
C14:0	11.10	12.79	0.254	0.012
C14:1 *cis*-9	1.03	1.04	0.035	0.782
C15:0	0.64	0.68	0.013	0.838
C16:0	34.57	37.41	0.653	0.024
C16:1 *cis*-9	1.20	1.28	0.035	0.485
C18:0	9.65	9.19	0.332	0.041
C18:1 *trans*-9	4.11	3.93	0.148	0.336
C18:1 *trans*-11	1.49	1.09	0.078	0.009
C18:1 *cis*-9	21.27	18.11	0.790	0.015
C18:2 *cis*-9,12	2.94	2.72	0.293	0.334
C18:2 *cis*-9, *trans*-11	0.151	0.112	0.007	<0.001
C18:2 *trans*-10, *cis*-12	0.120	0.125	0.008	0.636
C18:3n-3	0.324	0.267	0.008	0.031
C20:0	0.161	0.143	0.007	0.284
C20:3n-6	0.123	0.116	0.012	0.639
C22:0	0.040	0.039	0.011	0.594
C22:6n-3	0.041	0.038	0.005	0.492
*FA groups, % of total FA*				
Total SFA	67.20	71.17	0.753	0.014
Total MUFA	29.10	25.45	0.681	0.011
Total PUFA	3.70	3.378	0.169	0.521
Total CLA	0.271	0.237	0.010	0.133

1 FA, fatty acids; SFA, saturated fatty acids; MUFA, monounsaturated fatty acids; PUFA, polyunsaturated fatty acids; CLA, conjugated linoleic acids.

2 HS-free, heat stress-free; HS, heat stress.

In total, 767 lipid species were detected and quantified across all milk samples, with all lipids present in both the HS and HS-free groups. Based on the classification system from the LIPID MAPS consortium, these lipids were categorized into three major classes: glycerolipids (GL, 52.4 %), glycerophospholipids (GP, 35.3 %), and sphingolipids (SP, 12.3 %) (Table S6).

**Fig. 3:**
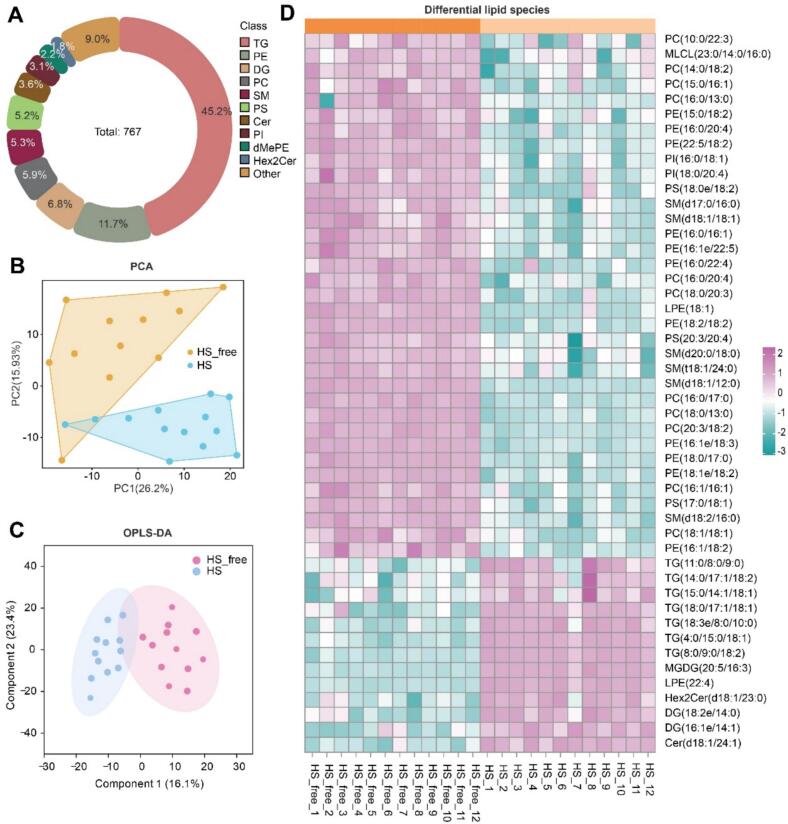
Lipidomic analysis of milk samples from HS and HS-free cows. (A) Donut chart showing the proportion of lipid subclasses in milk samples. (B) Principal component analysis (PCA) score plot of milk lipid specie. (C) Orthogonal partial least squares discriminant analysis (OPLS-DA) score plot of milk lipid profiles. (D) Heatmap of differential lipid species (VIP > 1.0 and *P* < 0.05) between HS-free and HS cows. Each row represents a lipid species, and each column represents a sample. Color scale represents standardized abundance (*Z*-score). TG: Triacylglycerol; DG: Diacylglycerol; MGDG: Monogalactosyldiacylglycerol; PE: Phosphatidylethanolamine; dMePE: Dimethylphosphatidylethanolamine; PC: Phosphatidylcholine; LPE: Lysophosphatidylethanolamine; PI: Phosphatidylinositol; PS: Phosphatidylserine; MLCL: Monolysocardiolipin; Cer: Ceramide; Hex2Cer: Dihexosylceramide; SM: Sphingomyelin; HS, heat stress; HS-free, heat stress-free; VIP, variable importance in projection.

Additionally, these lipids were classified into 25 subclasses (Fig. 3A), including triacylglycerol (TG, 45.2 %), phosphatidylethanolamine (PE, 11.7 %), diacylglycerol (DG, 6.8 %), phosphatidylcholine (PC, 5.9 %), sphingomyelin (SM, 5.3 %), phosphatidylserine (PS, 5.2 %), ceramide (Cer), phosphatidylinositol (PI), dimethylphosphatidylethanolamine (dMePE), dihexosylceramide (Hex2Cer), lysophosphatidylethanolamine (LPE), monolysocardiolipin (MLCL), hexosylceramide (Hex1Cer), bis-methyl phosphatidic acid (BisMePA), cardiolipin (CL), lysophosphatidylcholine (LPC), lysodimethylphosphatidylethanolamine (LdMePE), phosphatidic acid (PA), monogalactosyldiacylglycerol (MGDG), lysophosphatidylinositol (LPI), lysophosphatidylserine (LPS), phosphatidylethanol (PEt), sphingosine (SPH), sulfoquinovosyldiacylglycerol (SQDG), and phosphatidylglycerol (PG).

The PCA of the milk lipid profiles demonstrated a distinct separation between the HS and HS-free groups (Fig. 3B), indicating marked differences in lipid composition. To further identify discriminative features, OPLS-DA was employed, which confirmed a clear distinction between the two groups. These results highlight that heat stress substantially modifies the lipidomic landscape of milk. A total of 48 differential lipid species were identified (VIP > 1 and *P* < 0.05) between the HS-free and HS groups (Fig. 3D). Among them, 35 lipid species exhibited significantly lower abundance in the HS group, while 13 were increased. Notably, many of the downregulated lipids belonged to PE, PC, and SM, indicating that heat stress may impair the synthesis or secretion of membrane-associated lipids in milk.

### 3.6 Milk volatile compound profiles

The HS-SPME/GC–MS is widely employed in food flavor analysis, particularly for profiling volatile compounds in dairy products. As shown in Table S7, a total of 53 volatile compounds were identified in the milk samples. These compounds were categorized into several chemical classes: 10 acids, 5 alcohols, 4 alkanes, 2 alkenes, 9 aldehydes, 10 esters and ethers, 10 ketones, and 4 sulfur-containing compounds.

**Fig. 4:**
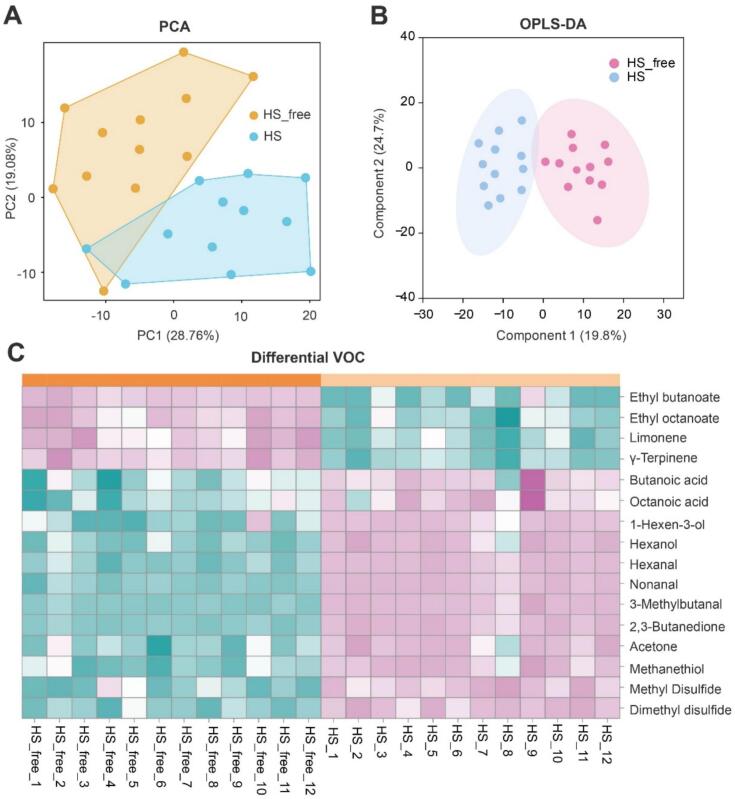
Multivariate analysis and heatmap of differential volatile organic compounds (VOC) between HS and HS-free milk samples. (A) Principal component analysis (PCA) score plot. (B) Orthogonal partial least squares discriminant analysis (OPLS-DA) score plot. (C) Heatmap of 16 differential VOC (VIP > 1 and *P* < 0.05). Color scale represents Z-score normalized abundance across samples. HS, heat-stressed group; HS_free, non-heat-stressed group; VIP, variable importance in projection.

To assess how heat stress influences milk volatile compound profiles, multivariate analyses were conducted. The PCA displayed a clear differentiation between the HS and HS-free groups based on their VOC patterns (Fig. 4A). This distinction was further supported by OPLS-DA results, which showed well-defined group separation and clustering (Fig. 4B), highlighting the significant alterations in volatile profiles induced by heat stress.

Based on VIP > 1 and *P* < 0.05, a total of 16 differential VOCs were identified between the HS and HS_free groups (Fig. 4C). Among them, 12 compounds were significantly increased under heat stress, including butanoic acid, octanoic acid, 1-hexen-3-ol, hexanol, hexanal, nonanal, 3-methylbutanal, 2,3-butanedione, acetone, methanethiol, dimethyl disulfide, and methyl disulfide. Conversely, four compounds were downregulated, including ethyl butanoate, ethyl octanoate, limonene, γ-terpinene.

## 4 Discussion

Global climate change is intensifying the frequency and severity of heat stress events, posing a serious challenge to dairy production. High ambient temperatures detrimentally affect cows' physiology, leading to reduced milk yield and impaired milk quality (Garner et al., 2017). Our multi-omics analyses provide compelling evidence that heat stress compromises multiple dimensions of bovine milk's nutritional quality and flavor.

### 4.1 Antioxidant properties

When the THI surpasses 72, dairy cows experience mild heat stress, which impairs nutrient digestion and utilization (Cowley et al., 2015). Concurrently, heat exposure triggers oxidative stress and inflammatory responses (Daddam et al., 2023). During this condition, the generation of reactive oxygen species exceeds the capacity of the cow's antioxidant system to neutralize them—particularly in lactating cows, where energy demands for synthesizing milk components are exceptionally high (Sun et al., 2017). Alberghina et al. (2024) reported that as THI rose, dairy cows showed significantly higher plasma oxidative markers and lower antioxidant defense. In the present study, antioxidant capacity was diminished in heat-stressed milk, as evidenced by declines in antioxidant indicators (e.g., T-AOC) and/or elevations in oxidative damage markers (e.g., we noted increased malondialdehyde, a lipid peroxidation marker). The results indicated that the oxidative imbalance in blood can translate to compromised antioxidant content in milk. Indeed, fat-soluble antioxidants (vitamins A and E) are transferred to milk via the blood; their decline during heat stress suggests that milk from heat-stressed cows likely contains lower levels of these nutritionally important antioxidants, thereby diminishing its nutritional value for consumers. Heat stress reduces the antioxidant capacity of milk, weakening its defense against ROS and making it more susceptible to oxidation and rancidity during storage. This oxidative stress can damage key milk components—lipids may produce off-flavors, and proteins may lose nutritional and functional quality (Yan et al., 2025). Our observed increase in oxidative damage markers supports these effects. Guo et al. (2021) also reported that heat-induced oxidative stress promotes mammary cell apoptosis and lowers milk protein synthesis. Overall, these findings suggest that heat stress accelerates milk quality deterioration through oxidative mechanisms.

### 4.2 Milk microbial changes

The milk microbiota plays a vital role in udder health, milk quality, and safety, and is sensitive to both environmental and nutritional factors. In our study, heat stress significantly altered the microbial composition of raw milk, characterized by increased abundances of several opportunistic or spoilage-associated taxa. At the phylum level, the predominant groups—Proteobacteria, Firmicutes, Bacteroidota, and Actinobacteria—remained consistent across samples consistent with previous findings in dairy cows (Ceciliani et al., 2024; Cremonesi et al., 2018; Zhao et al., 2022).

At the genus level, heat stress led to the enrichment of several bacterial genera associated with mastitis, spoilage, and inflammation, including *Stenotrophomonas*, *Pseudopedobacter*, *Escherichia-Shigella*, and *Sphingobacterium*. *Stenotrophomonas*, for example, has been implicated in clinical mastitis (Kuehn et al., 2013) and keratin degradation (Yamamura et al., 2002). *Escherichia-Shigella* spp., often used as markers of enteric contamination or gut leakage, were also increased under heat stress. The genus is linked to subclinical inflammation and lower milk quality in previous studies (Pang et al., 2018; Wang, Chen, et al., 2022).

Proper refrigeration of raw milk immediately after collection and throughout transport is essential to preserving its quality. Psychrotrophic bacteria, known for thriving at low temperatures, are major contributors to milk spoilage and pose a significant challenge to the dairy industry due to the economic losses they cause (Ji et al., 2024; Saha et al., 2024). Species of *Sphingobacterium* mainly exhibit lipolytic activity, although some strains exhibit proteolytic activity as well (Hantsis-Zacharov & Halpern, 2007). Furthermore, Gram-negative bacteria such as *Alcaligenes* spp. also cause the spoilage of dairy products (Hantsis-Zacharov & Halpern, 2007). Hence, the elevated levels of *Sphingobacterium* and *Alcaligenes* observed in heat-stressed cows may play a role in compromising the sensory quality of raw milk and its derived dairy products.

### 4.3 Milk metabolites

Heat stress induced pronounced changes in the milk metabolite profile, reflecting widespread metabolic perturbations in the cows. Untargeted metabolomics revealed alterations in multiple metabolic pathways, notably those related to energy metabolism (carbohydrates and tricarboxylic acid cycle intermediates), amino acid metabolism, and purine/pyrimidine metabolism.

**Energy-related metabolites** were clearly disrupted. We noted higher lactate and possibly pyruvate in milk from heat-stressed cows, which could be a consequence of altered rumen fermentation or muscle metabolism. The accumulation of lactate in milk may also originate from rumen microbes; interestingly, some gut microbiome-derived metabolites were identified among the discriminative compounds in heat-stressed milk (Tian et al., 2016). This suggests a link between heat stress, rumen microbial activity, and mammary metabolism. Additionally, we observed changes in TCA cycle intermediates: for example, citrate tended to increase in heat-stressed milk, whereas the downstream acid like fumarate was decreased. A similar pattern was reported in cows with subclinical ketosis (a metabolic stress condition), where milk citrate was higher and α-ketoglutarate and fumarate were lower compared to healthy cows (Zhao et al., 2023). Higher citrate in milk may reflect a reduction in its utilization for de novo fatty acid synthesis in the mammary gland (since heat stress often lowers fat synthesis, leaving more citrate unused in the mammary cytosol). Lower suggest a sluggish TCA cycle, possibly due to nutrient scarcity or a diversion of nutrients toward gluconeogenesis and ketogenesis (Rojas-Gómez et al., 2025). Collectively, these shifts in energy metabolites paint a picture of impaired energy status: the cows under heat were not able to fully oxidize metabolic fuels, resulting in partial metabolic products (ketones, lactate) spilling into milk. Such metabolic inefficiency is a hallmark of heat-stressed physiology and directly contributes to the reduced nutritional quality of milk (e.g., elevated ketones can impart off-flavors and are a sign of energy-deficient milk).

Heat stress led to a general decrease in the concentration of most free amino acids in milk, consistent with the observed reduction in milk protein percentage. This likely reflects impaired mammary amino acid uptake and reduced protein synthesis under thermal stress (Kaufman et al., 2018). Notably, creatine levels were elevated in heat-stressed milk, which may indicate increased muscle protein breakdown and energy demand. Creatine plays a critical role in cellular energy buffering and is often upregulated under physiological stress to maintain ATP homeostasis (Sadri et al., 2025). The reduction in free amino acid concentrations may compromise milk's nutritional and functional properties. Amino acids contribute to milk flavor precursors, buffering capacity, and biological activity; their decrease could negatively affect taste, bioavailability of essential nutrients, and the development of functional dairy products (Daniloski et al., 2022).

### 4.4 Milk lipid and fatty acid profiles

Heat stress negatively impacted the nutritional profile of milk fat, as evidenced by the reduced proportions of MUFA, including CLA, and a concurrent increase in SFA. UFAs, particularly CLA and oleic acid (C18:1 *cis*-9), are associated with health-promoting effects such as anti-inflammatory, anti-carcinogenic, and cardioprotective properties (Badawy et al., 2023). A decline in these components reduces the functional and nutritional value of milk for human consumption. In contrast, the elevated levels of SFAs—especially palmitic (C16:0) and stearic acids (C18:0)—may contribute to less favorable health outcomes, as excessive dietary intake of SFAs has been linked to increased cardiovascular risk (Brassard et al., 2017; Mazur et al., 2024). The shift toward a more saturated lipid profile under heat stress thus not only reflects metabolic inefficiency but also diminishes milk's appeal as a source of beneficial fatty acids.

Our lipidomics analysis revealed a marked reduction in key polar lipids—including PC, PE, and SM—in the milk of heat-stressed cows. These lipid classes are known to play crucial roles in milk quality, contributing to membrane structure, emulsification properties, and infant nutrition (de Silva et al., 2021; Lopez, 2011). PC and PE are major components of the milk fat globule membrane (MFGM), which supports the stability of fat emulsions and carries bioactive molecules important for gut and brain development (Ortega-Anaya & Jiménez-Flores, 2019; Sultan et al., 2021). SM, abundant in the MFGM, is involved in neuronal growth and immune modulation, particularly in early life nutrition (Schneider et al., 2019; Yuan et al., 2024). Therefore, the observed decline in these beneficial lipids under heat stress may compromise the functional and nutritional quality of milk, particularly in products aimed at infants or health-conscious consumers.

### 4.5 Milk flavor compounds

One of the most striking consequences of heat stress observed in this study was its effect on milk flavor compounds. Using volatile metabolite analysis (HS–SPME/GC–MS), we found that heat stress alters the spectrum of aroma compounds in milk, generally in a manner that could be detrimental to flavor quality. In heat-stressed milk, several off-flavor–associated volatiles were significantly higher, while some desirable flavor notes were diminished.

Our analysis revealed increased levels of aldehydes (e.g., hexanal), ketones (e.g., acetone, 2-butanone), and sulfur-containing compounds (e.g., dimethyl disulfide) in heat-stressed milk. These compounds are commonly associated with off-flavors such as rancidity, solvent-like notes, and sulfurous odors, and are often perceived even at low concentrations, potentially leading to consumer rejection (Cai et al., 2024; Jia et al., 2024; Ning et al., 2025). Hexanal, a marker of lipid oxidation, was elevated in heat-stressed milk, likely due to the combined effects of increased saturated fat and decreased antioxidant levels (Ince et al., 2024). Similarly, the rise in dimethyl disulfide may stem from microbial degradation of sulfur-containing amino acids, in line with our observation of increased abundance of microbiota (Song et al., 2021). These shifts suggest that both oxidative chemical changes and microbial activity contribute to off-flavor formation under heat stress. In contrast, desirable aroma compounds like ethyl butanoate and ethyl acetate, which impart sweet and fruity notes (Zhou et al., 2025), were reduced. This reduction could result from altered mammary metabolism or loss of microbial diversity needed for ester biosynthesis (Lei et al., 2025). The net effect is a flatter, less pleasant flavor profile.

From a dairy industry perspective, these findings underscore the importance of maintaining milk flavor quality during heat waves. Early detection of volatile markers like hexanal or dimethyl disulfide may serve as indicators of milk spoilage risk. Moreover, interventions such as dietary antioxidant supplementation or rapid cooling post-milking may help preserve flavor integrity and consumer acceptability.

## 5 Conclusions

This study comprehensively evaluates the impact of heat stress on the compositional and functional quality of bovine raw milk using multi-omics approaches. Our findings demonstrate that heat stress significantly reduces antioxidant capacity, alters milk microbiota by favoring spoilage-associated and potentially pathogenic taxa, and disrupts key metabolic pathways. Specifically, heat-stressed milk exhibited changes in energy and amino acid metabolism, along with a decrease in nutritionally important components such as UFA, CLA, and polar lipids (e.g., PC, PE, SM). Additionally, the flavor profile of the milk was adversely affected, with increased levels of off-flavor volatiles (e.g., hexanal, ketones, sulfur compounds) and a decrease in desirable esters. These alterations can potentially compromise both consumer acceptance and processing quality. These conclusions are firmly supported by the data presented in this study, which provides molecular-level evidence for understanding the physiological stress caused by heat in dairy cows. Our results offer important insights for quality assessment, the development of early warning indicators, and future research aimed at mitigating the impact of climate stress on dairy production.

## CRediT authorship contribution statement

**Yuchao Zhao:** Writing – review & editing, Writing – original draft, Funding acquisition, Conceptualization. **Fenghong Wang:** Methodology, Investigation. **Ying Wang:** Software, Investigation, Data curation. **Jian Tan:** Investigation. **Haoyu Niu:** Investigation. **Gang Guo:** Resources. **Luoyun Fang:** Software. **Linshu Jiang:** Funding acquisition, Conceptualization.

## Declaration of competing interest

The authors declare that they have no known competing financial interests or personal relationships that could have appeared to influence the work reported in this paper.

## Data Availability

Data will be made available on request.

## Appendix A Supplementary data

Supplementary material Image 1
