# Supplementary material for: Heat stress compromises nutritional quality and flavor of bovine raw milk: Evidence from multi-omics analyses
Source: Food Chem X. 2025 Dec 3;32:103361. doi: 10.1016/j.fochx.2025.103361 (PMC12723045; doi:10.1016/j.fochx.2025.103361)
Supplement: Supplementary file 1 — Supplementary material [file mmc1.docx]

**Table S1.** Characteristics of the Holstein dairy cows used in the study.

| Item | HS-free | HS | *P*-value |
| --- | --- | --- | --- |
| Number of cows | 12 | 12 | — |
| Parity | 3 | 3 | — |
| Lactation days | 141±7 | 137±5 | 0.62 |
| 305-day milk  yield (kg) | 110289±732.1 | 110391±579.2 | 0.83 |
| Body weight (kg) | 682.0±17.6 | 697.2±23.4 | 0.55 |

**Table S2.** Ingredients and chemical composition of the total mixed ration.

| Item | Contents |
| --- | --- |
| Ingredient (% of DM) | |
| Corn silage | 34.59 |
| Alfalfa hay | 11.85 |
| Oat hay | 4.33 |
| Ground Corn grain | 17.73 |
| Flaked corn | 3.98 |
| Soybean meal | 10.46 |
| Canola meal | 2.90 |
| Sugar beet pulp | 0.56 |
| Whole cottonseed | 7.12 |
| Fat powder^2^ | 1.33 |
| Mineral and vitamin mix^2^ | 3.87 |
| Molasses | 1.28 |
| Chemical composition (% of DM)^3^ |  |
| DM, % as fed | 51.43 |
| OM | 91.12 |
| CP | 17.03 |
| NDF | 33.85 |
| ADF | 16.70 |
| EE | 4.96 |
| Starch | 25.69 |
| NE_L_ (Mcal/kg of DM)^4^ | 1.66 |

^1^Bergafat, a saturated free fatty acid supplement (Berg+Schmidt GmbH & Co. KG, Hamburg, Germany).

^2^Formulated to contain (as-is basis) trace mineral mix, 0.95%; dry corn distillers grains with solubles, 44.9%; MgO (56% Mg), 7.8%; NaCl, 6.6%; vitamin ADE premix, 0.48%; limestone, 39.2%; and selenium premix, 0.07%. Ca, 14.3%; P, 0.38%; Mg, 4.57%; K, 0.46%; S, 0.39%; Zn, 1,218 mg/kg; Fe, 186 mg/ kg, Se, 6.93 mg/kg; Cu, 370 mg/kg; vitamin A, 272,000 IU/kg; vitamin D, 75,000 IU/kg; and vitamin E, 2,080 IU/kg.

^3^Analyzed values.

^4^Calculated according to NASEM (2021).

| Item^1^ | | HS-free^2^ | | | HS | | |
| --- | --- | --- | --- | --- | --- | --- | --- |
|  |  | Temp. (℃) | RH (%) | THI | Temp. (℃) | RH (%) | THI |
| Day7 | 06:00 | 12.5 | 38.8 | 55.9 | 23.1 | 75.3 | 71.4 |
|  | 14:00 | 18.3 | 32.9 | 62.3 | 34.5 | 55.8 | 85.4 |
|  | 22:00 | 14.0 | 45.5 | 57.7 | 28.0 | 70.3 | 78.5 |
| Day6 | 06:00 | 11.8 | 42.4 | 55.4 | 22.5 | 78.2 | 70.8 |
|  | 14:00 | 17.5 | 39.1 | 61.9 | 35.4 | 50.7 | 85.0 |
|  | 22:00 | 13.5 | 45.2 | 57.1 | 29.1 | 72.4 | 80.3 |
| Day5 | 06:00 | 12.2 | 47.8 | 55.3 | 24.2 | 76.5 | 73.0 |
|  | 14:00 | 18.5 | 32.1 | 62.0 | 36.6 | 52.5 | 86.8 |
|  | 22:00 | 14.2 | 43.4 | 57.9 | 30.4 | 74.3 | 82.1 |
| Day4 | 06:00 | 12.0 | 43.0 | 55.2 | 23.5 | 77.9 | 72.3 |
|  | 14:00 | 17.4 | 34.5 | 62.2 | 35.0 | 53.0 | 86.3 |
|  | 22:00 | 13.6 | 38.7 | 57.5 | 29.1 | 73.1 | 81.2 |
| Day3 | 06:00 | 11.6 | 40.6 | 54.8 | 22.2 | 80.7 | 70.2 |
|  | 14:00 | 17.2 | 39.3 | 61.6 | 34.6 | 56.4 | 84.9 |
|  | 22:00 | 13.0 | 43.3 | 56.5 | 28.5 | 75.0 | 79.9 |
| Day2 | 06:00 | 12.4 | 44.8 | 55.7 | 24.8 | 74.5 | 73.6 |
|  | 14:00 | 18.8 | 30.2 | 63.1 | 36.7 | 54.8 | 87.9 |
|  | 22:00 | 14.5 | 46.7 | 58.3 | 30.5 | 71.4 | 82.4 |
| Day1 | 06:00 | 12.7 | 41.8 | 56.5 | 23.8 | 75.4 | 72.6 |
|  | 14:00 | 19.9 | 36.0 | 63.6 | 35.8 | 52.2 | 86.5 |
|  | 22:00 | 14.0 | 38.7 | 57.7 | 29.4 | 73.0 | 81.7 |

**Table S3.** Temperature and relative humidity for the dairy cows used in the study.

^1^Day 7, day 6, day 5, day 4, day 3, day2, and day 1 are respective time points before sample collection of dairy cows.

^2^Temp., temperature; RH, relative humidity. THI = (1.8×Temp. + 32) – (0.55 – 0.0055 × RH) × (1.8×Temp. – 26)).

**Table S4.** Rectal temperature and respiration rate of dairy cows.

| Item | | HS-free | HS | *P*-value |
| --- | --- | --- | --- | --- |
| Rectal temperature (  ℃) | 06:00 | 38.15±0.06 | 39.20±0.08 | < 0.01 |
|  | 14:00 | 38.47±0.08 | 39.56±0.09 | < 0.01 |
|  | 22:00 | 38.30±0.07 | 39.39±0.08 | < 0.01 |
| Respiration rate (breath  s/min) | 06:00 | 43.0±3.2 | 72.0±4.2 | < 0.01 |
|  | 14:00 | 49.1±3.6 | 76.3±4.7 | < 0.01 |
|  | 22:00 | 44.8±3.3 | 73.1±4.1 | < 0.01 |

**Table S5.** Lactational performance of dairy cows.

| Item | | HS-free | | HS | | *P*-value | |
| --- | --- | --- | --- | --- | --- | --- | --- |
| DMI, kg/d | | 24.9±1.8 | | 18.8±1.6 | | < 0.01 | |
| Milk yield, kg/d | | 36.8±2.5 | | 28.1±2.0 | | < 0.01 | |
| Milk composition |  | |  | |  | |  |
| Fat, % | | 4.25±0.18 | | 4.39±0.16 | | 0.34 | |
| Protein, % | | 3.28±0.12 | | 2.89±0.13 | | < 0.01 | |
| Lactose, % | | 4.95±0.11 | | 4.89±0.14 | | 0.85 | |
| MUN, mg/dL | | 13.8±0.9 | | 11.4±0.8 | | 0.01 | |
| SCC, ×10^3^ cells/mL | | 115.4±21.5 | | 372.3±45.1 | | < 0.01 | |

**Table S6.** Characterized lipid classes and number of lipid species.

| Lipid categories | Lipids classes | Abbreviation | Number of Species |
| --- | --- | --- | --- |
| Glycerolipids | Triacylglycerol | TG | 347 |
|  | Diacylglycerol | DG | 52 |
|  | Monogalactosyldiacylglycerol | MGDG | 2 |
|  | Sulfoquinovosyldiacylglycerol | SQDG | 1 |
| Glycerophospholipids | Bis-methyl phosphatidic acid | BisMePA | 8 |
|  | Cardiolipin | CL | 7 |
|  | Dimethylphosphatidylethanolamine | dMePE | 17 |
|  | Lysodimethylphosphatidylethanolamine | LdMePE | 3 |
|  | Lysophosphatidylcholine | LPC | 7 |
|  | Lysophosphatidylethanolamine | LPE | 11 |
|  | Lysophosphatidylinositol | LPI | 2 |
|  | Lysophosphatidylserine | LPS | 2 |
|  | Monolysocardiolipin | MLCL | 9 |
|  | Phosphatidylcholine | PC | 45 |
|  | Phosphatidylethanolamine | PE | 90 |
|  | Phosphatidylinositol | PI | 24 |
|  | Phosphatidylserine | PS | 40 |
|  | Phosphatidic acid | PA | 3 |
|  | Phosphatidylethanol | PEt | 2 |
|  | Phosphatidylglycerol | PG | 1 |
| Sphingolipids | Ceramide | Cer | 28 |
|  | Hexosylceramide | Hex1Cer | 9 |
|  | Dihexosylceramide | Hex2Cer | 14 |
|  | Sphingomyelin | SM | 41 |
|  | Sphingosine | SPH | 2 |

**Table S7.** Volatile compounds detected in the headspace of raw milk flour with HS-SPME/GC-MS.

| Number | Compound name | CAS no. |
| --- | --- | --- |
|  | **Acids** |  |
| 1 | Ethyl(nitro)carbamic acid | 219542-23-1 |
| 2 | Prop-2-enoic acid | 202326-54-3 |
| 3 | 2-Oxoacetic acid | 298-12-4 |
| 4 | Acetic acid | 64-19-7 |
| 5 | Butanoic acid | 107-92-6 |
| 6 | Pentanoic acid | 64118-37-2 |
| 7 | Octanoic acid | 124-07-2 |
| 8 | Decanoic acid | 334-48-5 |
| 9 | Tetradecanoic acid | 544-63-8 |
| 10 | Lauric acid | 143–07-7 |
|  | **Alcohols** |  |
| 11 | Ethanol | 64-17-5 |
| 12 | Hexanol | 111-27-3 |
| 13 | 2-Ethylhexanol | 104-76-7 |
| 14 | 1-Pentanol | 71-41-0 |
| 15 | 1-Hexen-3-ol | 4798-44-1 |
|  | **Alkanes** |  |
| 16 | Dodecane | 112-40-3 |
| 17 | Hexane | 10-54-3 |
| 18 | Toluene | 108-88-3 |
| 19 | Tetradecane | 629–59-4 |
|  | **Alkenes** |  |
| 20 | Limonene | 138-86-3 |
| 21 | γ-Terpinene | 99-85-4 |
|  | **Aldehydes** |  |
| 22 | Furfural | 98-01-1 |
| 23 | Benzaldehyde | 100-52-7 |
| 24 | Hexanal | 66-25-1 |
| 25 | Heptanal | 111-71-7 |
| 26 | 3-Methylbutanal | 590-86-3 |
| 27 | 5-Methyl furfural | 620-02-0 |
| 28 | Nonanal | 124-19-6 |
| 29 | Octanal | 124-13-0 |
| 30 | Pentanal | 110-62-3 |
|  | **Esters and Ethers** |  |
| 31 | Bis(2-Cyanoethyl)Ether | 1656-48-0 |
| 32 | Dimethyl ether | 115-10-6 |
| 33 | Methyl acetate | 79-20-9 |
| 34 | Ethyl acetate | 141-78-6 |
| 35 | Ethyl caproate | 123-66-0 |
| 36 | Butyl acetate | 123-86-4 |
| 37 | Ethyl butanoate | 105-54-4 |
| 38 | Ethyl hexanoate | 123-66-0 |
| 39 | Ethyl octanoate |  |
| 40 | Propyl acetate | 109-60-4 |
|  | **Ketones** |  |
| 40 | Acetone | 67-64-1 |
| 41 | 2-Butanone | 78-93-3 |
| 42 | 2,3-Butanedione | 431-03-8 |
| 43 | 2-Pentanone | 107-87-9 |
| 44 | 3-Pentanone | 96-22-0 |
| 45 | 2-Cyclopentenone | 930-30-3 |
| 46 | Cyclopentanone | 120-92-3 |
| 47 | 2-Heptanone | 110-43-0 |
| 48 | Acetophenone | 98-86-2 |
| 49 | 3-Hydroxy-2-butanone | 513-86-0 |
|  | **Sulfur** |  |
| 50 | Methyl Disulfide | 624-92-0 |
| 51 | Methanethiol | 74-93-1 |
| 52 | Dimethyl disulfide | 624-92-0 |
| 53 | Dimethyl sulfone | 667-71-0 |


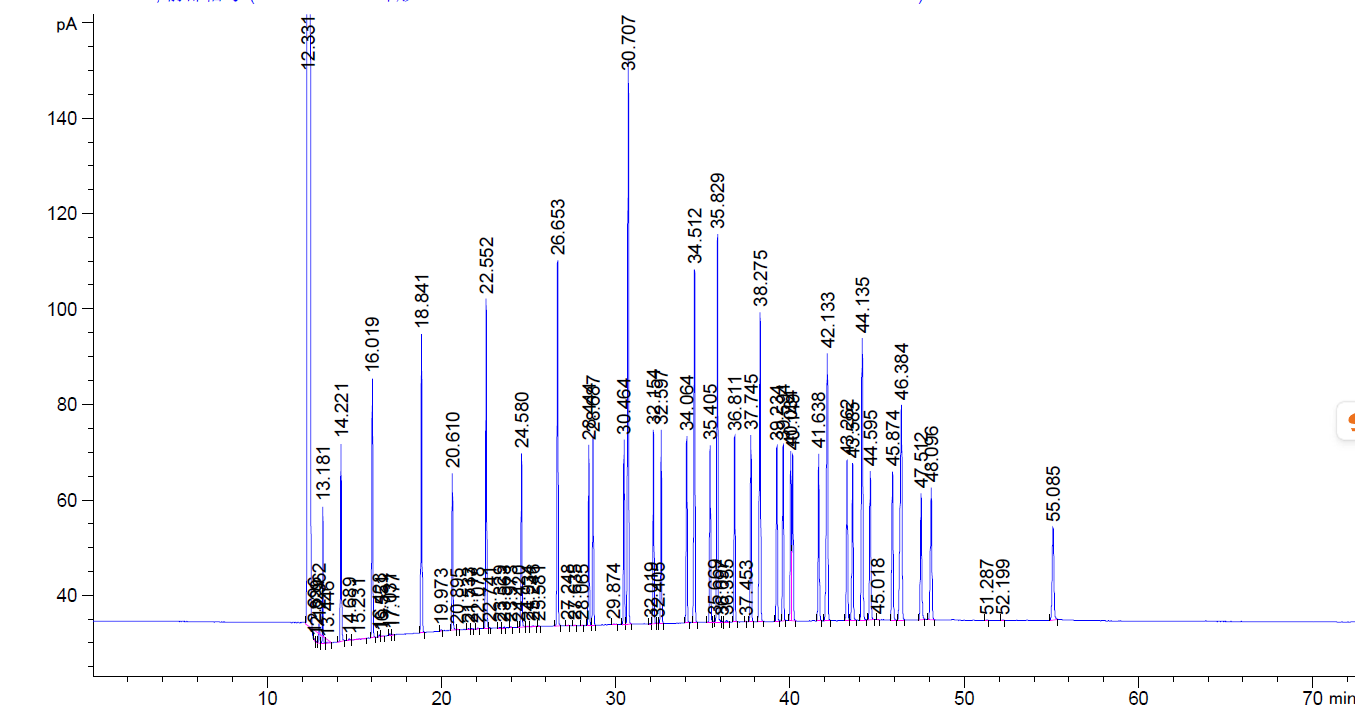


Figure S1. A chromatographic profile of milk fatty acids.


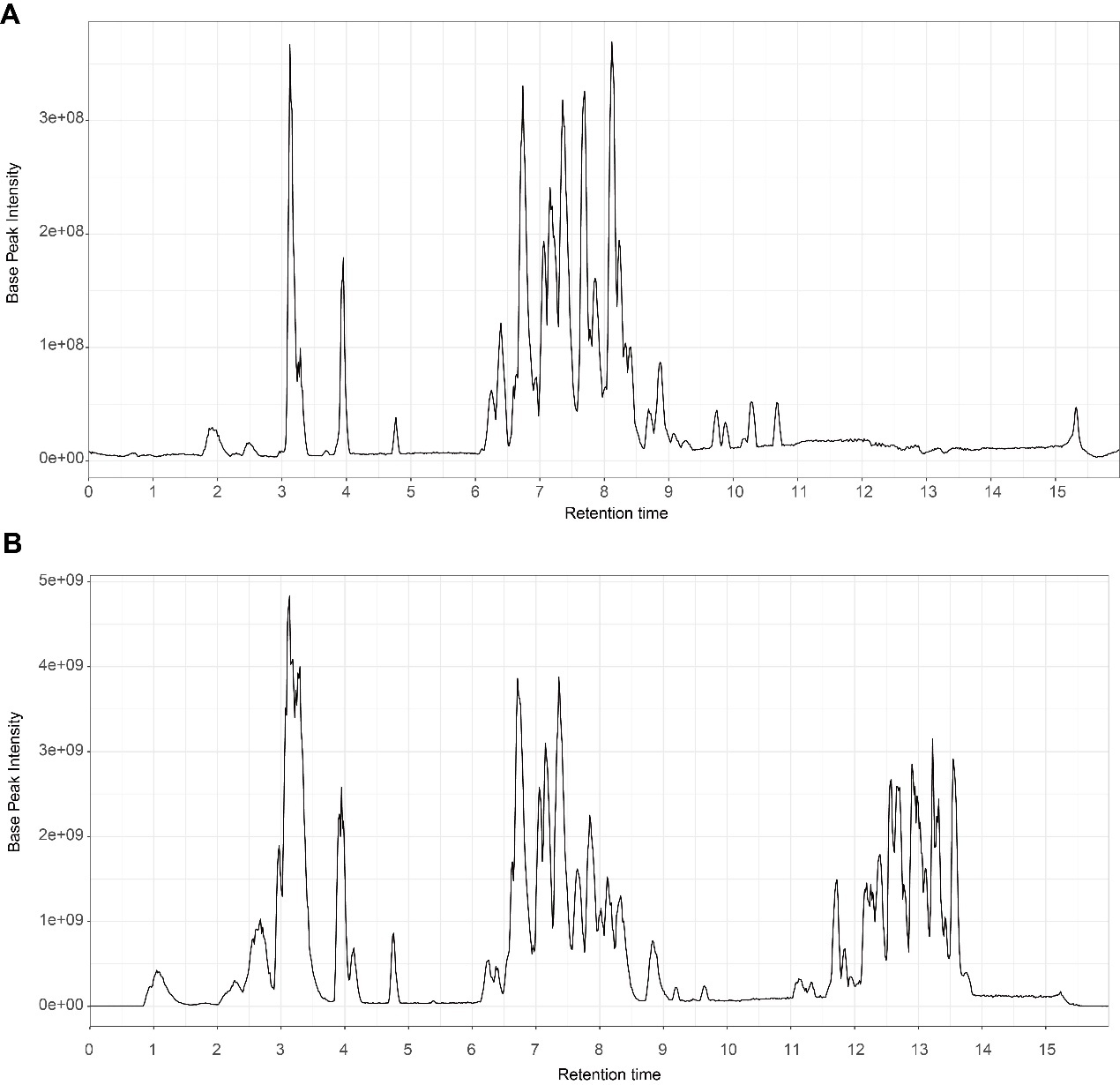


Figure S2. Examples of chromatograms for milk lipidomics. (A) Negative model and (B) Positive model.


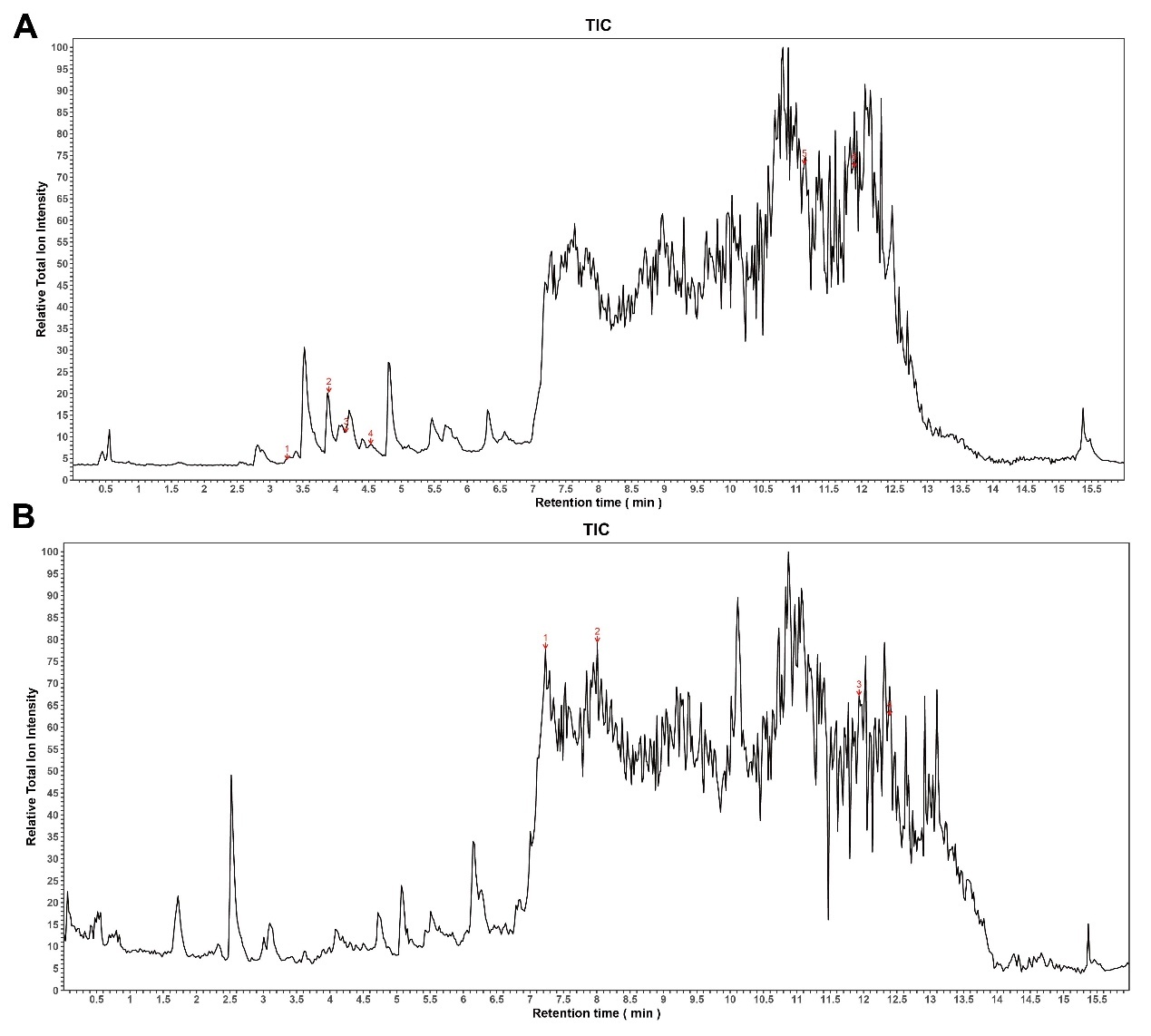


Figure S3. Examples of chromatograms for LC-MS-based metabolomics. (A) Negative model and (B) Positive model.


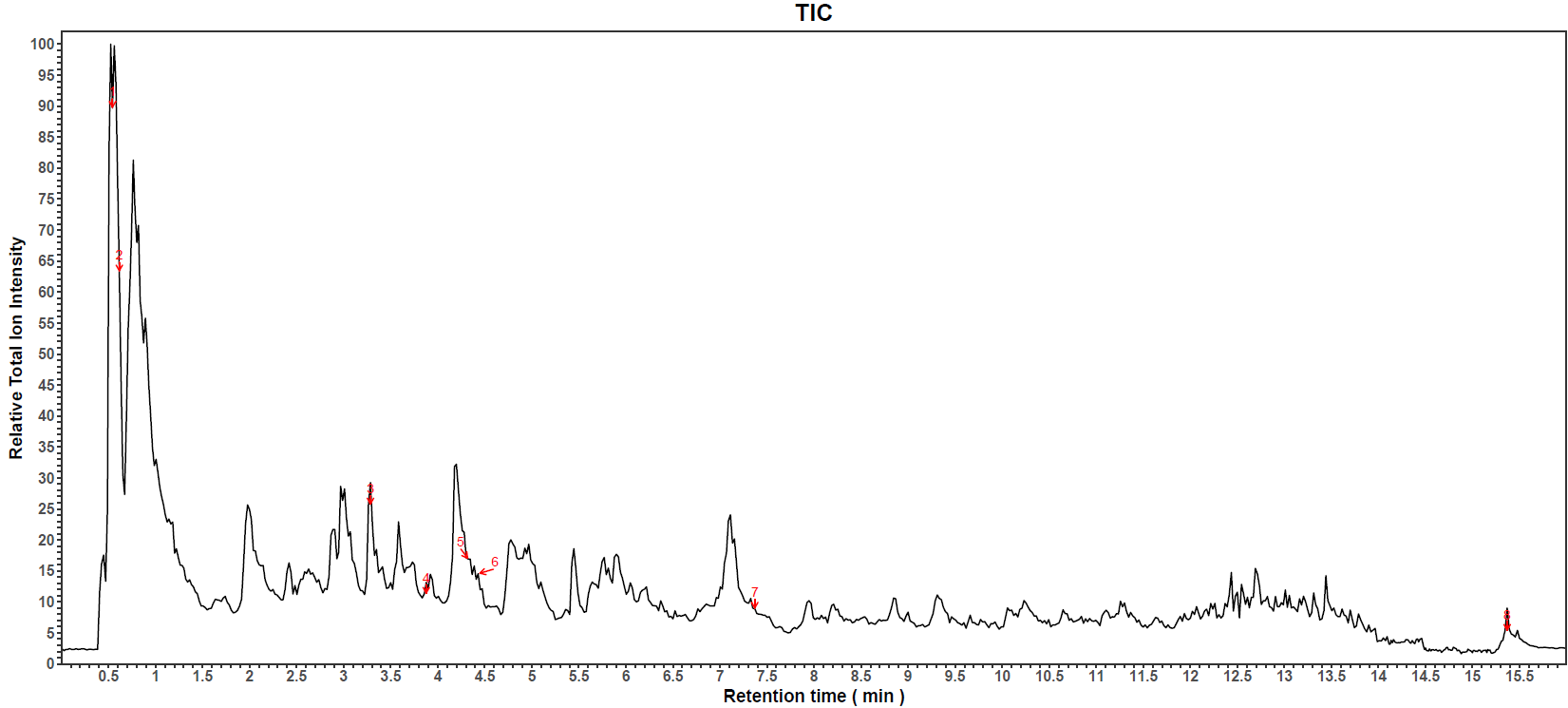


Figure S4. A chromatographic profile of milk volatile metabolomics.
